# Estimation of mRNA COVID-19 Vaccination Effectiveness in Tokyo for Omicron Variants BA.2 and BA.5: Effect of Social Behavior

**DOI:** 10.3390/vaccines10111820

**Published:** 2022-10-28

**Authors:** Sachiko Kodera, Yuki Niimi, Essam A. Rashed, Naoki Yoshinaga, Masashi Toyoda, Akimasa Hirata

**Affiliations:** 1 Department of Electrical and Mechanical Engineering, Nagoya Institute of Technology, Nagoya 466-8555, Japan; 2 Center of Biomedical Physics and Information Technology, Nagoya Institute of Technology, Nagoya 466-8555, Japan; 3 Graduate School of Information Science, University of Hyogo, Kobe 650-0047, Japan; 4 Institute of Industrial Science, the University of Tokyo, Tokyo 153-8505, Japan

**Keywords:** COVID-19, vaccination effectiveness, Omicron variant, Twitter

## Abstract

The variability of the COVID-19 vaccination effectiveness (VE) should be assessed with a resolution of a few days, assuming that the VE is influenced by public behavior and social activity. Here, the VE for the Omicron variants (BA.2 and BA.5) is numerically derived for Japan’s population for the second and third vaccination doses. We then evaluated the daily VE variation due to social behavior from the daily data reports in Tokyo. The VE for the Omicron variants (BA.1, BA.2, and BA.5) are derived from the data of Japan and Tokyo with a computational approach. In addition, the effect of the different parameters regarding human behavior on VE was assessed using daily data in Tokyo. The individual VE for the Omicron BA.2 in Japan was 61% (95% CI: 57–65%) for the second dose of the vaccination from our computation, whereas that for the third dose was 86% (95% CI: 84–88%). The individual BA.5 VE for the second and third doses are 37% (95% CI: 33–40%) and 63% (95% CI: 61–65%). The reduction in the daily VE from the estimated value was closely correlated to the number of tweets related to social gatherings on Twitter. The number of tweets considered here would be one of the new candidates for VE evaluation and surveillance affecting the viral transmission.

## 1. Introduction

The emergence of COVID-19 in December 2019 resulted in significant mortality worldwide [1]. Because of the development of new vaccines and high vaccination rates, the pandemic was temporarily controlled in some countries [2]. However, a resurgence has been observed in the latter half of 2021, in European countries, and in Japan in early 2022, for example. This would be primarily due to the higher infectivity of the Delta and Omicron variants [3] and waning vaccination immunity [4]. Reduced vaccination effectiveness (VE) has also been extensively reported for the Omicron variant [5]. Moreover, some countries have adopted no restrictions following the wide spread of variants in 2022.

In general, two measures are used to characterize vaccination related to waning immunity; efficacy and effectiveness [6]. Vaccine efficacy, which is determined via randomized controlled trials (typically clinical trials), is defined as vaccine protection measured under optimal conditions, where vaccine storage and delivery are monitored, and participants are usually healthy. Conversely, vaccine effectiveness assesses how well a vaccine performs in real-world settings [7]. Thus, vaccination efficacy overestimates the actual effectiveness. There are several mechanisms and factors that influence the efficacy. One significant issue is time-dependent antibody reduction, which has been linked to vaccination efficacy [8]. The neutralizing antibody kinetics were reported 6 months after a full vaccination with BNT162b2 [9]. For healthcare workers, time-dependent Nabs reduction, antibodies against the nucleocapsid, and the SARS-CoV-2 spike protein were measured [10]. Observational and computational studies suggested that antibody reduction over time is a predictor of immune protection from a symptomatic infection [11,12].

The effectiveness or real-world performance is rather divergent in different countries [6,13,14,15]. Higher protection levels against COVID-19 infection for the Delta variant were reported in a cohort study [13]; the individual VE was 93% (95% CI: 85–97%) in the first month after vaccination, but declined to 53% (95% CI: 39–65%) after four months. The meta-analysis mean value for a systematic review of the Delta variant was 60.5% and 75.6% for partial and full vaccination, respectively [16]. In [17], the individual VE against a symptomatic infection for the Delta variant was 89% (95% CI: 86–92%) 7–59 days after a second dose, but declined to 80% (95% CI: 74–84%) after ≥240 days. It increased to 97% (95% CI: 96–98%) ≥7 days after a third dose. Instead, the VE against a symptomatic Omicron infection was 36% (95%CI: 24–45%) 7–59 days after a second dose and almost no protection after ≥180 days. The VE then increased to 61% (95% CI: 56–65%) ≥7 days after a third dose. In [18], the individual VE for the Omicron variants BA.1 and BA.2 were almost the same against a symptomatic disease, whereas 25 weeks or more after two doses, the individual VE was 14.8% against BA.1 and 27.8% against BA.2. Booster immunization increased one’s protection after a week to 70.6% against BA.1 and 74.0% against BA.2, waning to 37.4% for BA.1 and 43.7% for BA.2 at 15 or more weeks after receiving the booster dose. One possible explanation for these differences is the amount of viral exposure, which is influenced by human behavior. Some vaccines would protect against infection from small viral exposures but not from large exposures [19]. The additional correlated factors that represent public activities may exist [20]. However, due to socio-physiological differences, these factors are known to have varying correlations with the spread of the pandemic. We deduced that the waning immunity of vaccination is the individual VE for the Delta and Omicron variants in our previous study [21]: for the second dose, the individual VE for the Delta and Omicron BA.1 variants (two weeks after the 2nd shot) were estimated as 93.8% (95% CI: 93.1–94.6%) for the whole Japanese population and 62.1% (95% CI: 48–66%) for the population in Tokyo, respectively.

The gap between the efficacy and effectiveness as well as the variability of different countries would be attributable to daily behavior (e.g., mask wearing and social gathering). Most observatory studies were evaluated in terms of elapsed time after full vaccination (second dose) or a booster shot (third dose). The observation time resolution was a few weeks to months (e.g., 2 weeks, 2, 4, and 6 months after vaccination), in addition to the different vaccination timing for each individual. However, behavioral change may be observed in a time resolution of a few hours to several days, e.g., superspreading events (social gathering) for the former and New Year holidays, etc., which are often characterized by the three Cs (closed spaces, crowded places, and close contact). Thus, the real-world VE reported in observatory studies can be considered as the time-averaged value for such combined factors. An additional factor is the population VE from the transmission of viral variants, which is closely related to herd immunity [22,23] or the antibody prevalence ratio [24]. It would be helpful to discuss prevention measures as well as individual VE variability in different countries if the population VE can be estimated in a resolution of a few days.

In this study, the waning VE immunity has been evaluated for the whole population of Tokyo from 23 December 2021 to 5 March 2022, for the Omicron BA.1 variant and that in Japan from 11 April 2022 to 31 July 2022, for the Omicron variants BA.2 and BA.5. The waning effect and behavior change in the VE for infection prevention has been numerically estimated for the population in Japan for the second and third doses. We particularly considered the number of tweets associated with the risk of infection as one of the metrics.

## 2. Materials and Methods

### 2.1. Study Design

We have conducted two computational experiments to derive the VE. Here, we define the vaccine effectiveness as a proportion, using the following formula: VE = 1 − relative risk [25]. The first experiment is the derivation of individual VE for the BA.1, BA.2, and BA.5 variants derived from the Japanese population using weekly data. The other is the investigation of the daily behavioral factors that characterize the daily reduction in the individual VE in Tokyo. As the input data, three metrics were considered: (i) the mobility at the transit stations, (ii) the keywords of Twitter (social drinking, BBQ, and Karaoke), and (iii) the nighttime population who stayed in the downtown area, including in restaurants and bars.

Among the vaccinated population, approximately 90% of individuals were vaccinated with the Pfizer BNT162b2; the remaining were vaccinated with the mRNA-1273 Moderna COVID-19 vaccine for their first and second shots in Japan. Similarly, Pfizer BNT162b2 (58.8%) and mRNA-1273 Moderna (41.1%) are mainly employed, in addition to Novavax (0.1%) [26]. Due to the low Novavax percentage, the two mRNA vaccines are considered jointly assuming that their VE is almost the same. Tokyo started to report the classification of new daily positive cases into “2nd (or more) vaccinated” or “unvaccinated” [27]. Note that in the third doses, Moderna and Pfizer were vaccinated regardless of the manufacturer of the first and second doses.

### 2.2. Computational Approach

In [21], we proposed the computational method to estimate the waning immunity of vaccination among the Japanese population. The statistical data needed for this approach were the daily number of vaccinated individuals and daily positive unvaccinated cases and fully vaccinated individuals (second dose). This has been extended to treat the third and fourth doses.

(1) $$e_{t}\left(i\right)=\{\begin{array}{c} a_{t}\cdot i/K\;\left(i\leq K\right),\\ a_{t}-s\left(i-K\right)\;\left(i>K\right), \end{array}$$

where *e_t_* (*i*) is the VE on the *i*th days after the inoculation for the *t*th dose (= 1–4), and the parameters of *a* and *s* were adjusted to reach a peak *K* days after the inoculation (*K* = 14 for the second dose and *K* = 7 for the third and fourth dose), then decrease linearly. The individual VE for the first shot was assumed to be constant after 14 days due to a lack of data. As shown in Equation (1), a waning immunity is expressed as linear with time. This is consistent with the tendency of vaccination efficacy in [12] where the neutralizing antibody is reduced with the time almost linearly. Thus, for the same vaccine, this parameter *s* was kept as 0.15 (95% CI: 0.13–0.17) as in the second dose.

**Figure 1:**
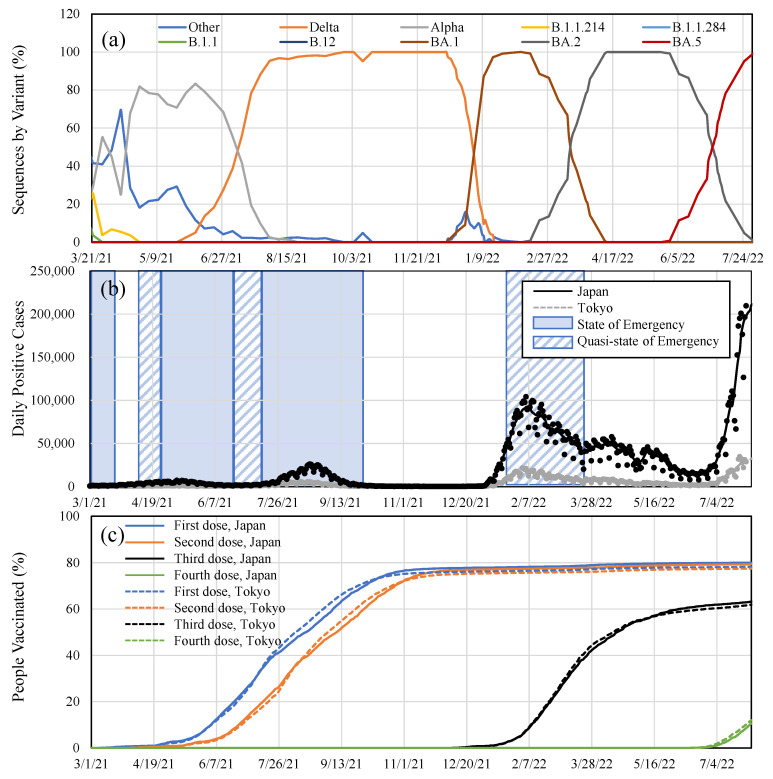
Time course of (**a**) SARS-CoV-2 sequences by variants in Tokyo. Time courses of (**b**) daily positive cases and (**c**) vaccination rate in Tokyo and over Japan.

The population VE is required to estimate the effective unprotected population from infection. The population VE *E* (*d*) was assumed to be as follows:

(2) $$E\left(d\right)=\overset{T}{\underset{t=1}{\sum }}\overset{d}{\underset{i=0}{\sum }}\left(N_{t}\left(d-i\right)\cdot v\left(i\right)e_{t}\left(i\right)\right)/P,\;$$

where *d* is the day index and *N_t_* denotes the number of people who were newly administrated a vaccination dose (*t* = 1–4). *P* is the population of Japan (126,645,025 people) or Tokyo (13,843,329 people). The *v* denotes the SARS-CoV-2 sequences by variants, as shown in Figure 1a. The waning effect was overwritten when people took the second or third dose by adjusting the number of people vaccinated in the past [28].

An in-house Python code was used to calculate the population VE. The optimal parameter *a_t_* for each variant was derived by comparing the COVID-19 prevalence among unvaccinated people and without immunity to prevent infection. The weekly number of people without immunity to prevent infection was calculated from the 7-day average of the daily population VE. The root mean squared percentage error (RMSPE) of that prevalence was used as an evaluation index (RMSPE < 10%).

### 2.3. Data for Vaccination

Vaccination in Japan began in March 2021, first for medical workers, then elderly individuals, and then in June 2021 for non-medical workers, stretching almost uniformly across the country. The interval between the first and second shots was controlled at 3 to 4 weeks. The third and fourth shot was then started from 1 December 2021 and 25 May 2022, respectively. The daily number of people who were newly administrated a vaccination used in Equation (2) are available from the Vaccination Record System (VRM) by the Digital Agency, Japan [29].

The data needed for fitting the parameter *a* for the *t*th dose in Equation (1) are from the website of the Ministry of Health, Labor, and Welfare [30] of Japan and the press release by the Tokyo Metropolitan Government [27]. The dataset in [30] includes the weekly number of unvaccinated, fully vaccinated individuals, and those vaccinated with a booster dose, for the number of infected individuals in each category from 11 April 2022 to 31 July 2022. The Tokyo dataset for the daily information [27], which is available only for Tokyo (population 13.9 million), will be used to discuss the effect of our behavior on the VE. The overall age categories are not given for the Tokyo data. However, waning immunity is comparable to the age category >65 and <65 years in Japan (less than 2% after the second shot) [21]. Subjects without information regarding vaccination were excluded from this study (approximately 30%) [31]. The time slot from 11 April 2022 to 25 May 2022, and that from 13 July 2022 to 7 August 2022, were used for estimating the VE for the Omicron variants BA.2 and BA.5, respectively. These correspond to the period >80% occupancy for each variant (See Figure 1a). The effective vaccination of the fourth shot reached less than 5%, considering a lag of one-week of effectiveness at the end of July 2022; the VE for the fourth shot assumed to be marginal.

The SARS-CoV-2 sequences by variants in Tokyo are shown in Figure 1. Note that the sequence for Japan is not available. Additionally, the number of daily positive cases and the percentage of people vaccinated are shown in the same figure.

### 2.4. Data Associated with Social Behavior

We considered three metrics to assess the effect of social behavior on the individual VE: (i) mobility at the transit stations, (ii) nighttime population who stayed in the downtown area, including restaurants and bars [32], and (iii) Twitter keywords (social gathering for drinking and a BBQ). These are the human behavior metrics made available by the Cabinet Secretariat COVID-19 AI Simulation Project (https://www.covid19-ai.jp/en-us/ accessed on 2 September 2022). The rationale for these metrics is based on a study [33] that found that most infections can be traced back to specific locations, particularly restaurants. If these metrics can explain the daily variation in VE, they would be excellent candidates for long-term monitoring.

The mobility data [34] were often used as a metric for a viral transmission, whereas it may not always be relevant to the 3 Cs as it may not clearly indicate the social behavior of the population. We then considered the latter two metrics in addition to mobility.

The nighttime population who stayed in the area near restaurants and bars was obtained from NTT DOCOMO, INC, also accessible under the project of the Cabinet Secretariat COVID-19 AI Simulation Project, Japan.

**Figure 2:**
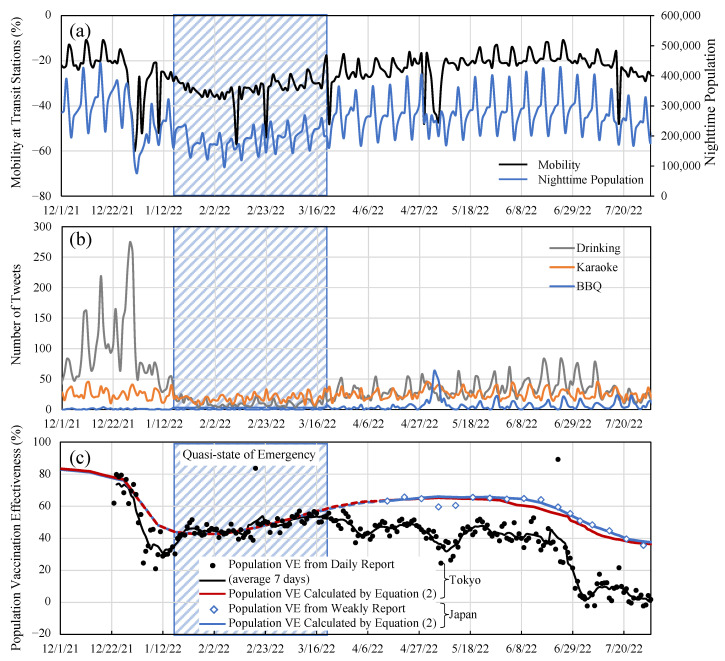
Time course of (**a**) mobility at transit stations and nighttime population volume in downtown area of Tokyo, (**b**) the number of tweets for social drinking and BBQ, and (**c**) Population VE estimated from daily report in Tokyo (dashed lines) and that weekly reported over Japan (solid lines).

Twitter data were used as it may indicate social activities where close contact had occurred. The downtown population was considered as several domestic reports have indicated that the main infection clusters may be due to close contacts in these areas (see Figure 2).

Twitter data were obtained from the NTT Data, INC. and processed by Toyoda Lab., University of Tokyo, and shared through the Cabinet Secretariat COVID-19 AI Simulation Project. Tweeted keywords completed during the day or the previous day or those planned until the next day were extracted when determining the number of tweeted keywords. The recorded data can clearly indicate the time frames where these events were more popular, although it is difficult to confirm if these gathering events were actually held or not. For corresponding tweets, information on the prefecture was extracted from the user’s address.

A 7-day average value is considered for considering the weekly effect when discussing the correlation with VE. An 8- and 5-day time lag of three behavior metrics is also considered on New Year holidays and golden week, respectively, which corresponds to the incubation and delay until the report for the Delta and Omicron variants [35].

### 2.5. Statistical Analysis

To assess the effect of human behavior on the population VE, the statistical analysis was carried out using Python 3.9.7. A linear regression model was used to fit the drop from the estimated population VE as the dependent variables for each independent variable: the number of tweets, the nighttime population, and the mobility at the transit stations. The Spearman’s rank correlation test was also carried out. The threshold for a statistical significance was set at *p* < 0.05.

## 3. Results

### 3.1. Vaccination Effectiveness for BA.2 and BA.5

In Table 1, the parameters of the Omicron BA.2 and BA.5 variants were derived for the Japanese population using the procedure described in Section 2.2. According to Table 1, the individual VE of the BA.2 variant just after the third shot (*a*_3_) was 86%, which was higher than the 61% for the second shot. For the BA.5 variant, the estimated value was lower than that for the BA.2 variant. Due to lack of data for Japan, the parameters of the Omicron BA.1 variant were also derived from the Tokyo data: *a*_2_ = 63 (95% CI: 61–64) and *a*_3_ = 85 (95% CI: 80–90). The parameters of the *a*_2_ and *a*_3_ value for BA.2 were comparable to those of BA.1.

As shown in Figure 2c, the population’s VEs over Japan (weekly) and Tokyo (daily) were calculated by substituting the parameters derived from Table 1 into Equation (2). The VE calculated from the value reported by Tokyo is also plotted in the same figure. The reported population VE was in a good agreement with the estimated VE during the quasi-state of emergency, whereas some drops (reduction) in the reported VE from the estimated value were observed during a specific period. Similar tendencies were observed between the VE for the population over Japan and that in Tokyo. The former was 10–15% higher than that of the latter averaged over 1 week from April to May (BA.2 variant), whereas it was 25–30% in July (BA.5 variant). A clear decay is observed in some of the periods, unlike in the 1-week averaged value.

### 3.2. Effect of Different Factors on Vaccination Effectiveness in Tokyo

**Figure 3:**
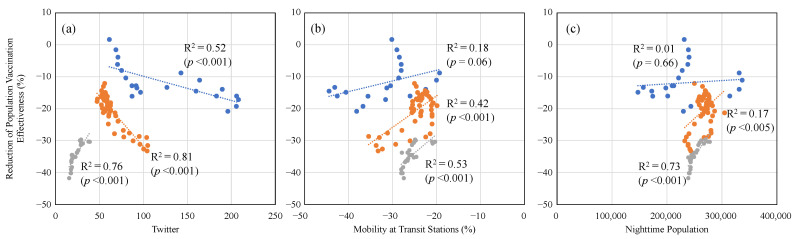
Correlation between 7-day average daily drop from estimated population VE and (**a**) Twitter, (**b**) mobility at transit stations, (**c**) nighttime population before and after the quasi-state of emergency. The period of blue plots, orange plots, and gray plots are from 24 December 2021 to 13 January 2022, from 24 March 2022 to 14 May 2022, and form 15 July 2022 to 31 July 2022, respectively.

Figure 3 shows the correlation between the 7-day average daily drop in the reported population VE from the estimated VE and different factors (mobility, Twitter, and the nighttime population). The summation of tweets related to drinking, karaoke, and a BBQ were used as an index for Twitter. A strong correlation has been observed between them, as shown in Figure 3. The slopes are different before and after the quasi-state of emergency. Table 2 displays the Spearman’s rank correlation for each period. As for the number of tweets, a single keyword “social drinking” was considered (not shown in Figure 3), as well as a combination of three keywords (“social drinking,” “BBQ,” and “Karaoke”). From March to May 2022, correlation was higher for the three keywords rather than for the single keyword of social drinking.

## 4. Discussion

In this study, we numerically derived the parameters characterizing the individual VE for the Omicron variants: BA.1 for the population in Tokyo of 13.8 million and BA.2 and BA.5 for the population in Japan of 126.6 million. The feature of our approach extended here based on our previous study, [21], is that from the data of a limited observatory; the individual VE can be estimated with a simple computation. In [21], the parameters characterizing the VE, which are derived for the data from 11 January 2022 until 24 January 2022, worked well until early January.

We compared the result in the cohort study in Tokyo and the suburb area [36]: the suggested individual VE for the Delta and Omicron-dominant period was 88% (95% CI: 82–93) and 56% (95% CI: 37–70) for 14 days to 3 months after a second dose, confirming our numerical derivation. The individual VE averaged over 14 days to 3 months was 88% and 53%, figures which are in an excellent agreement with our computational model.

The individual VE for the BA.2 and BA.5 periods was derived for the Japanese population. The population VE was derived and compared with the observed data in Tokyo. The individual VE of a second dose BA.1 in Tokyo and BA.2 in Japan was 63% (95%CI: 61–64) and 61% (95%CI: 57–65) after 14 days. From Equation (2), the estimated individual VE for BA.1 and BA.2 after 25 weeks was 37%, which is in a fair agreement with 14.8% against BA.1 and 27.8% against BA.2 [18]. In addition, the third dose VE was 85% (95% CI: 80–90) and 86% (95%CI: 84–88) for BA.1 and BA.2, respectively. These values were 70.6% and 74.0%, respectively, in [18].

A slightly smaller population VE was observed than that of the theoretical value, except during the quasi-state of emergency. As shown in Figure 3, the number of tweets, which is related to an infection risk, is correlated with the decrease in the vaccination effectiveness. Unlike the Twitter data, a correlation was observed, but it was not strong, for mobility and the nighttime population. This hypothesized that the sentiment to the writer’s behavior on Twitter may reflect the decrease in the VE rather than the physical number of phenomena, which may not directly lead to an infection if an appropriate countermeasure was taken. In Figure 3 and Table 2, we have shown the combined data including social drinking, karaoke, and a BBQ (*ρ* = −0.83 [*p* < 0.001] and *ρ* = −0.80 [*p* < 0.001] before and after the quasi-state of emergency, respectively). The correlation of the reduction in the population VE with Twitter only for social drinking decreased after the quasi-state of emergency (*ρ* = −0.83 [*p* < 0.001] and *ρ* = −0.36 [*p* < 0.001] before and after the quasi-state of emergency, respectively). It is worth noting that the percentage of the younger population (aged in their 20s and 30s) at night was 53%, which increased after the quasi-state emergency (see Appendix A). In Twitter, the users’ demographic data, such as the age and gender, are not available and thus are not used here.

Some drop in the reported population VE from the estimated population VE was observed during the New Year and golden week (in early May) holidays, in addition to a heat wave, with a time lag of approximately 5 days. In particular, a substantial decrease in the theoretical value was observed from the period of 28 June to 15 July 2022, which approximately corresponds to the national elections (from 23 June to 10 July 2022). The tendency in July was different from other periods. The bottom appeared on around 5 July 2022, which potentially relates to the heat wave when the maximum daily ambient temperature was above 35 °C. This out-of-season heat wave may cause less air ventilation.

The ratio of people who have immunity by infection was marginal because of the number of people infected with the Delta and Omicron variants until December 17 was less than 1.5% of the total Tokyo population and 0.7% of Japan, respectively. We considered that the people infected without symptoms would be four times higher than that of the reported cases [37]. The impact on the individual VE is marginal before the end of June. However, that effect may influence the VE or immunity during the seventh wave where the BA.5 variant was predominant. However, its impact may not be negligible in the seventh wave (beginning in late June) because 14% of the unvaccinated population was infected; when non-symptomatic infection is considered, the figure rises to more than 70%.

The population VE during the quasi-state of emergency, when such tweets are almost nonexistent, would be a good metric for vaccination efficacy. The population VE in Tokyo is lower than that over Japan, which could be attributed to the population density [38,39]. Further, the population density has played a role in different countries [40].

In summary, the VE for mRNA COVID-19 vaccines was calculated using limited data for the entire Japanese population. In Omicron subvariants, its effectiveness was lower than in previous variants. The population VE was discovered to vary over time, which could be partly attributable to our behavior. The number of tweets related to some risk on Twitter, i.e., the keywords (“social drinking,” “BBQ,” and “Karaoke”), was found to correlate with a decrease in the VE. The reduction in the VE had a significant impact on viral transmission [21]. Thus, the surveillance or monitoring of tweets would be a crucial factor for forecasting the spread of COVID-19 [28].

## Figures and Tables

**Table 1:** Parameters for the individual VE of second and third shots used in Equation (1) for Omicron variants. For comparison, the parameters for parameters in Tokyo derived in our previous study [16] are also shown.

		1st Shot (*a*_1_)	2nd Shot (*a*_2_)	3rd Shot (*a*_3_)
Delta [16]	Mean	75	96	
(Japan)	95% CI	(71–79)	(96–97)	
Omicron (BA.2) (Japan)	Mean		61	86
	95% CI		(57–65)	(84–88)
Omicron (BA.5) (Japan)	Mean		37	63
	95% CI		(33–40)	(61–65)
Omicron (BA.1) (Tokyo)	Mean		63	85
	95% CI		(61–64)	(80–90)

**Table 2:** Spearman’s rank correlation between 7-day average daily drop from estimated population effectiveness of vaccination and different parameters of human behavior.

		Twitter (Three Keywords)	Twitter (Social Drinking)	Nighttime Population	Mobility
24 December 2021–13 January 2022	*ρ*	−0.83	−0.83	0.16	0.54
	*p*-value	<0.001	<0.001	0.47	0.01
24 March 2022–14 May 2022	*ρ*	−0.80	−0.36	0.16	0.35
	*p*-value	<0.001	<0.001	0.47	0.01
15 July 2022–31 July 2022	*ρ*	0.92	0.93	0.93	0.79
	*p*-value	<0.001	<0.001	<0.001	<0.001

**Figure A1:**
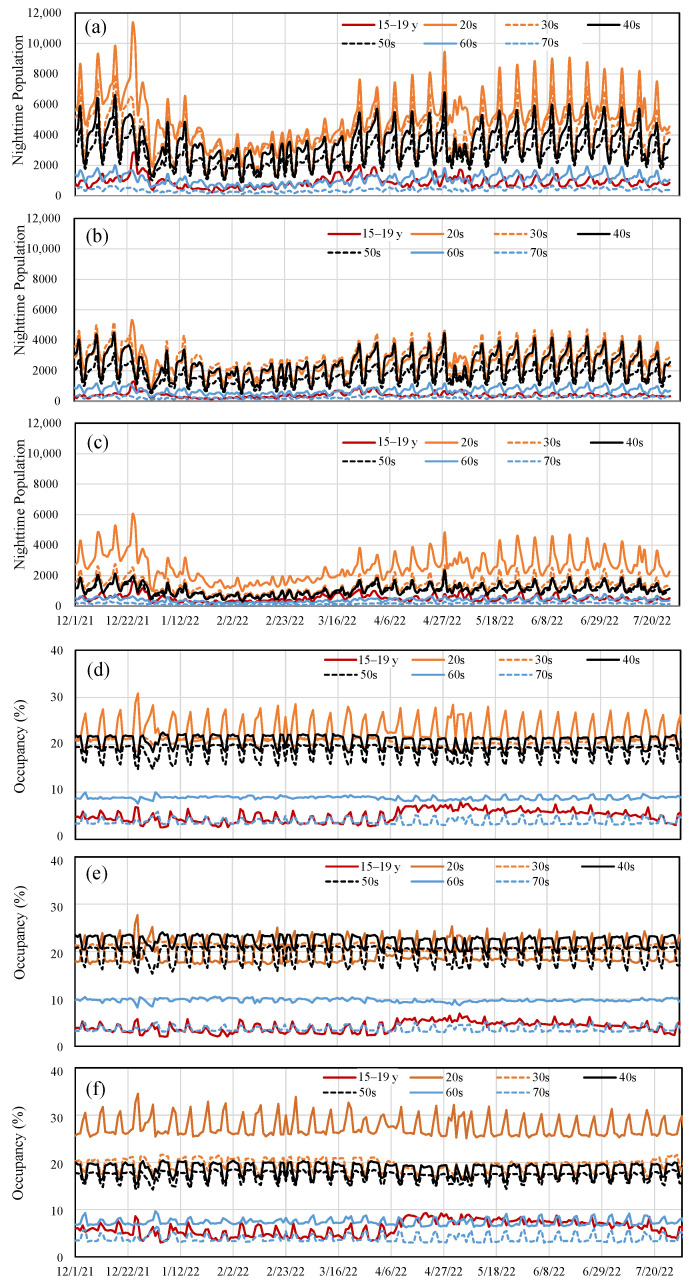
(**a**–**c**) Time course of the nighttime population by age category and (**d**–**f**) the occupancy of age category in the mobility at the station. The gender categories are (**a**,**d**) all, (**b**,**e**) male, and (**c**,**f**) female.

## Data Availability

Data (except for Twitter) processed in this study will be available with reasonable request, ending 5 years following article publication.

## Appendix A

Figure A1 depicts the time course of the nighttime and daytime population and mobility at transit stations near downtown, classified by age. As illustrated in Figure A1, the majority of the population is comprised of younger individuals. The nighttime population is dominated by people in their 20s. Moreover, the mobility at transit stations is dominated by people in their 20s to 50s.
